# Animal Welfare Attitudes: Effects of Gender and Diet in University Samples from 22 Countries

**DOI:** 10.3390/ani11071893

**Published:** 2021-06-25

**Authors:** Christoph Randler, Ana Adan, Maria-Mihaela Antofie, Arturo Arrona-Palacios, Manecas Candido, Jelle Boeve-de Pauw, Priti Chandrakar, Eda Demirhan, Vassilis Detsis, Lee Di Milia, Jana Fančovičová, Niklas Gericke, Prasun Haldar, Zeinab Heidari, Konrad S. Jankowski, Juhani E. Lehto, Ryan Lundell-Creagh, William Medina-Jerez, Adrian Meule, Taciano L. Milfont, Mireia Orgilés, Alexandra Morales, Vincenzo Natale, Xóchitl Ortiz-Jiménez, Babita Pande, Timo Partonen, Atanu Kumar Pati, Pavol Prokop, Arash Rahafar, Martin Scheuch, Subhashis Sahu, Iztok Tomažič, Lorenzo Tonetti, Pablo Vallejo Medina, Peter van Petegem, Alejandro Vargas, Christian Vollmer

**Affiliations:** 1 Department of Biology, University of Tuebingen, Morgenstelle 24, D-72076 Tuebingen, Germany; 2 LEAD Graduate School and Research Network, University of Tübingen, D-72072 Tübingen, Germany; 3 Department of Biology, Faculty of Natural Sciences and Technology, University of Education Heidelberg, D-69120 Heidelberg, Germany; 4 Department of Clinical Psychology and Psychobiology, School of Psychology, University of Barcelona, 08035 Barcelona, Spain; aadan@ub.edu; 5 Institute of Neurosciences, University of Barcelona, 08035 Barcelona, Spain; 6 Faculty of Agricultural Sciences, Food Industry and Environmental Protection, “Lucian Blaga” University of Sibiu, 550012 Sibiu, Romania; mihaela.antofie@ulbsibiu.ro; 7 Writing Lab, Institute for the Future of Education, Tecnologico de Monterrey, 64849 Monterery, Mexico; a.arrona@hotmail.com; 8 Department of Natural Sciences, Universidade Pedagogica Mazombique, University Rovuma, 3100 Nampula, Mozambique; manecascandido@yahoo.com; 9 Department of Training and Education Sciences, University of Antwerp, Prinsstraat 13, 2000 Antwerp, Belgium; jelle.boevedepauw@uantwerpen.be (J.B.-d.P.); peter.vanpetegem@uantwerpen.be (P.v.P.); 10 SoS in Life Science, Pt. Ravishankar Shukla University, Raipur 492010, India; pritichandrakar229@gmail.com (P.C.); babitatime14@gmail.com (B.P.); akpati19@gmail.com (A.K.P.); 11 Department of Special Education, Sakarya University, Sakarya 54300, Turkey; edemirhan@sakarya.edu.tr; 12 Department of Economics and Sustainable Development, Harokopio University, Venizelou 70, 17676 Athens, Greece; detsis@hua.gr; 13 School of Business & Law, CQ University Australia, Rockhampton, QLD 4701, Australia; v.dimilia@cqu.edu.au; 14 Department of Biology, Faculty of Education, Trnava University, Priemyselná 4, 918 43 Trnava, Slovakia; jana.fancovicova@truni.sk; 15 Department of Environmental and Life Sciences, Karlstad University, 65188 Karlstad, Sweden; niklas.gericke@kau.se; 16 Department of Biological Sciences, Midnapore City College, Paschim Medinipur 721129, West Bengal, India; ssprasun0@gmail.com; 17 Independent Researcher, 1653676331 Tehran, Iran; veganlife2012@gmail.com (Z.H.); ar.ra.rhythm@gmail.com (A.R.); 18 Faculty of Psychology, University of Warsaw, 00-183 Warszawa, Poland; kjankows@psych.uw.edu.pl; 19 Educational Sciences, Open University, P.O. Box 9 (Siltavuorenpenger 3 A), University of Helsinki, 00014 Helsinki, Finland; juhani.e.lehto@helsinki.fi; 20 Department of Psychology, Bishops University, Sherbrooke, QC J1M 1Z7, Canada; RLUNDELL12@ubishops.ca; 21 College of Education, University of Texas at El Paso, El Paso, TX 79968, USA; wjmedinajerez@utep.edu; 22 Department of Psychiatry and Psychotherapy, University Hospital of the LMU Munich, Nußbaumstraße 7, 80336 Munich, Germany; ameule@med.lmu.de; 23 Schoen Clinic Roseneck, Am Roseneck 6, 83209 Prien am Chiemsee, Germany; 24 School of Psychology, University of Waikato, 3240 Hamilton, New Zealand; taciano.milfont@waikato.ac.nz; 25 Department of Health Psychology, Miguel Hernández University, 03202 Elche (Alicante), Spain; morgiles@umh.es (M.O.); alexandra.moraless@umh.es (A.M.); 26 Department of Psychology “Renzo Canestrari”, University of Bologna, Viale Berti Pichat 5, 40127 Bologna, Italy; vincenzo.natale@unibo.it (V.N.); lorenzo.tonetti2@unibo.it (L.T.); 27 School of Psychology, Autonomous University of Nuevo Leon, 64460 Monterrey, Mexico; xortizj@gmail.com; 28 Department of Public Health and Welfare, Finnish Institute for Health and Welfare (THL), P.O. Box 30 (Mannerheimintie 166), 00271 Helsinki, Finland; timo.partonen@thl.fi; 29 Center for Translational Chronobiology, Pt. Ravishankar Shukla University, Raipur 492010, India; 30 Gangadhar Meher University, Sambalpur 768004, India; 31 Department of Environmental Ecology and Landscape Management, Faculty of Natural Sciences, Comenius University, Ilkovičova 6, 842 15 Bratislava, Slovakia; pavol.prokop@savba.sk; 32 Institute of Zoology, Slovak Academy of Sciences, Dúbravská cesta 9, 845 06 Bratislava, Slovakia; 33 Austrian Educational Competence Centre for Biology, University of Vienna, Porzellangasse 4/2, 1090 Vienna, Austria; martin.scheuch@univie.ac.at; 34 Environmental Education, University College for Agricultural & Environmental Education, Angermayergasse 1, 1130 Vienna, Austria; 35 Ergonomics & Occupational Physiology Laboratory, Department of Physiology, University of Kalyani, Kalyani 741235, West Bengal, India; sahuphysiolku@gmail.com; 36 Department of Biology, Biotechnical Faculty, University of Ljubljana, Večna pot 111, 1000 Ljubljana, Slovenia; iztok.tomazic@bf.uni-lj.si; 37 Fundación Universitaria Konrad Lorenz. Cra. 9 Bis #62-43, 110231 Bogotá, Colombia; pablo.vallejom@konradlorenz.edu.co (P.V.M.); alejandro.vargasc@konradlorenz.edu.co (A.V.); 38 Department of Research and Development in Teacher Education, University College of Teacher Education Tyrol, Pastorsstr. 7, 6020 Innsbruck, Austria; christian.vollmer@ph-tirol.ac.at

**Keywords:** animal welfare attitudes, diet, gender, gender inequality, culture

## Abstract

**Simple Summary:**

Animal Welfare Attitudes (AWA) can be defined as the attitudes of humans towards the welfare of animals. Although AWA has been previously associated with demographic factors as gender, one of the main limitations is that few studies applied robust psychometric questionnaire scales. Moreover, some evidence of cross-cultural variations in AWA have been reported although limited by the reduced number of countries being examined. To overcome these limitations, a survey aimed at assessing the gender differences in AWA in university students living in 22 nations, based on a questionnaire having undergone psychometric testing (i.e., the Composite Respect for Animals Scale Short version, CRAS-S), was carried out. To this end, the CRAS-S was administered to 7914 people (5155 women, 2711 men, 48 diverse) alongside a questionnaire on demographic information and diet. Moreover, the gender inequality index, based on indicators as completion of secondary education, was computed. The main results showed that diet was significantly related to AWA; more in detail, higher AWA was observed in vegans compared to omnivores. Moreover, gender differences in AWA have been reported, with women referring higher AWA compared to men. In addition, to the decreasing of gender inequality, gender differences in AWA increased.

**Abstract:**

Animal Welfare Attitudes (AWA) are defined as human attitudes towards the welfare of animals in different dimensions and settings. Demographic factors, such as age and gender are associated with AWA. The aim of this study was to assess gender differences among university students in a large convenience sample from twenty-two nations in AWA. A total of 7914 people participated in the study (5155 women, 2711 men, 48 diverse). Participants completed a questionnaire that collected demographic data, typical diet and responses to the Composite Respect for Animals Scale Short version (CRAS-S). In addition, we used a measure of gender empowerment from the Human Development Report. The largest variance in AWA was explained by diet, followed by country and gender. In terms of diet, 6385 participants reported to be omnivores, 296 as pescatarian, 637 ate a vegetarian diet and 434 were vegans (*n* = 162 without answer). Diet was related with CRAS-S scores; people with a vegan diet scored higher in AWA than omnivores. Women scored significantly higher on AWA than men. Furthermore, gender differences in AWA increased as gender inequality decreased.

## 1. Introduction

The protection and treatment of animals has increasingly gained public attention and the concept of animal welfare is complex and multi-dimensional [1]. Another barrier to understanding how humans feel, think and care about animals is a number of different constructs, definitions and measurements. We here view attitudes toward animal welfare as a broad psychological construct of attitude. In this respect, animal welfare is a useful umbrella term for several dimensions regarding human attitudes toward animals.

Demographic factors, such as gender, age, educational level, or socioeconomic status are associated with Animal Welfare Attitudes [2,3]. Previous work across many countries has revealed gender differences with girls and women expressing higher pro-animal welfare attitudes than boys and men in many studies, review papers and in a meta-analysis of the literature [2,4,5,6,7,8,9,10].

Based on an undergraduate sample, found that males showed lower AWA than females [6]. Drawing on samples in the USA, Japan and 13 European countries, focused on the attitudes towards the use of animals for research purposes [7]. Their results suggested women were more sensitive concerning animal welfare compared to men. In a cross-cultural comparison, showed that female university students had a greater concern for animal welfare rights than males [11]. Women were more concerned with the welfare of animals (pigs, laying hens) and were more supportive towards more restrictive animal welfare legislation [12]. Finally, in a meta-analysis showed that gender differences vary according to the amount of human animal-interaction [4]. Gender differences were greater in animal activism, recreational hunting and animal cruelty, of medium concern towards animal use and animal hoarding, and small concerning animal attachment as pets [4].

Age is another demographic factor associated with the acceptance of the use of animals in research [13]. This relationship exhibits a curvilinear pattern [14] suggesting that AWA become more positive from childhood to adolescence, but afterwards, AWA become less favorable [14,15,16,17]. Not all studies have identified a distinct age effect [18]. A recent review identified that attitudes toward farm animals decreased with age, i.e., older people were less concerned about farm animal welfare [2]. In addition, it might also be a cohort effect which influences AWA. Our study was mainly based on university students, so age was mainly used as a covariate (statistical control).

Lifestyle factors such as diet are also implicated in AWA. Recent studies have reported a negative relationship between AWA and meat consumption [15,19,20] and in some studies, the coefficients albeit significant were small [20]. It has been reported that of the vegetarians among university students the proportion of females is three times higher than that of the male students [11]. Therefore, diet is incorporated in our analysis.

The literature has also identified cross-cultural differences. On one hand, with reference to human basic traits it can be assumed that humans behave in similar ways across cultures (i.e., universality), on the other hand the adoption and practice of attitudes and behavior are greatly modulated by cultural diversity.

One of the first large cross-cultural studies (*n* = 3432) in AWA, carried out by in 11 countries across Europe and Asia, reported that female students had greater concerns for animal welfare and rights than males, especially in more gender empowered countries [11]. Another limitation of the cross-sectional literature is the absence of taking into account the social and economic development of the countries in considering AWA (but see [21]). Therefore, we will focus on gender differences with respect to different socioeconomic development. In another study, Sinclair and Phillips investigated 13 major world social issues in 12 nations and found that animal and environmental protection and sustainable development were the most highly rated in importance across all countries [22]. Thus, socioeconomic development must be considered.

Another major limitation is that few studies have applied robust psychometric questionnaire scales. For example, Von Roten asked their sample only two questions about the use of monkeys and mice in health research across Europe and found a general reluctance towards the use of monkeys for research purposes [23]. Therefore, our study was based on a questionnaire having undergone psychometric testing, i.e., the Composite Respect for Animals Scale Short version (CRAS-S; [24]).

Ling et al., further emphasized that AWA have been extensively studied for people in developed countries, but there are few studies from the developing countries [25]. This is important because animal production might be different in countries with a different socio-economic development. For example, Sinclair et al., showed that nationality was the most important predictor influencing attitude during slaughter and transport in stakeholders in SE and E Asia [26]. In contrast, most cross-cultural studies have compared two or three countries. Therefore, we moved forward by studying more than just a handful of countries.

### Current Study

In this study, AWA are defined as human attitudes towards the welfare of animals in different dimensions and settings. These dimensions stretch from utilitarian aspects such as using animals for food, clothing, recreation, and research to concerns many individuals have about pets, conservancy of species, and attitudes of superiority over animals [13,14,15,24]. Thus, our study is not restricted to farm animal welfare. We address gender differences taking large samples from diverse countries across six continents to establish universality. In addition, we used age as a statistical control (covariate) and diet as some external validation. We hypothesize that women should score higher on AWA, and that a vegan/vegetarian diet should be related with higher scores in AWA. Further, a country’s gender inequality index might be related to gender differences in AWA.

## 2. Materials and Methods

### 2.1. Procedure

Data were collected in 2016 based on a convenience sample of undergraduate university students from 22 countries, across all continents and translated into 23 languages. Most of the data is drawn from European countries. In total, we obtained 24 samples. In each of the 22 countries, a collaborator contacted participants asked them to voluntarily complete the questionnaire via a web-link or by paper pencil in classrooms. In Spain, the questionnaire was completed in Barcelona and in Elche, both in Spanish. In India, the questionnaire was presented in English and Hindi. Survey administration lasted between five and ten minutes. Thus, there were 24 samples with 23 translations and 22 countries. In each country, the questionnaires were collected in specific universities or specific cities (affiliations of the researchers). See details in Table 1. Data sampling was based on a convenience sampling method–previous collaborators in our studies were invited to participate in this study, and if they disagreed, asked for further possible collaborators, as well as for ‘snowball’ sampling by asking them to provide this information to their collaborators. Further, we tried to recruit colleagues via Researchgate and by direct e-mail. No country was excluded, but the bias was towards Europe.

**Figure 1:**
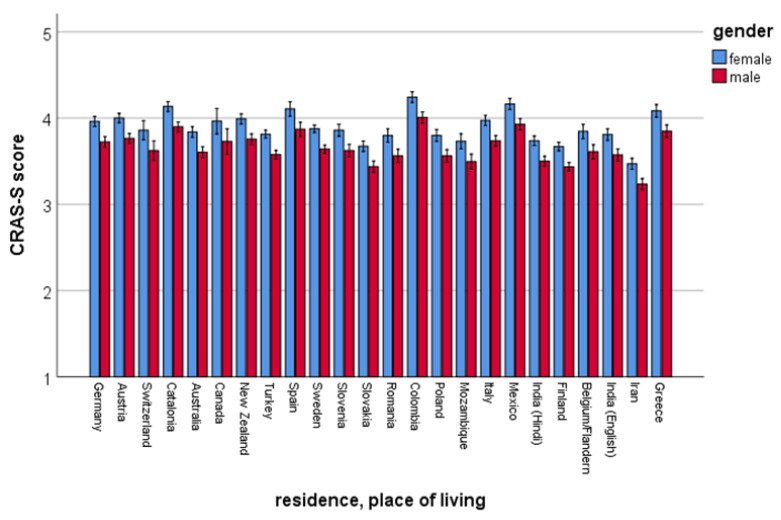
Gender differences according to countries (based on the estimated marginal means derived from the general linear model). Higher scores represent higher pro-animal welfare attitudes. Animal welfare attitudes (AWA) were measured with the Composite Respect for Animals Scale-Short Version (CRAS-S).

In the general linear model, diet explained about 11% of the variance, residence 10% and gender 4% of the variance. The effect sizes were medium for diet and residence, and small for gender (Table 2). Women scored significantly higher on AWA than men (estimated marginal means (± SE): women: 3.89 ± 0.013 versus men: 3.66 ± 0.015). The distribution of gender across country/residence is shown in Figure 1.

The collaborators were instructed to administer an anonymous survey among at least 200 participants with a good balance in genders (50% women, 50% men). The student samples were based on a convenience sampling method. Most of the samples were within this expectation (Table 1). Some countries (e.g., Switzerland and Canada) did not reach this target but were included. Many collaborators collected a higher sample. The original scale items were in English. The questionnaire was translated into the official language of the respective country or region where the study was carried out. The scales were translated by native speakers.

### 2.2. Questionnaire

Participants completed a questionnaire that included demographic data and the Composite Respect for Animals Scale Short version [24]. AWA has been assessed with a variety of questionnaire scales that tapped into several constructs and dimensions. The CRAS-S was developed as a ‘composite’ of some scales [15], to create a 20-item scale that measures a broad construct of AWA including the use of animals for research, food production, and clothing. The scale can be found in Appendix A. Responses to the CRAS-S are recorded on a five-point Likert-type format ranging from 1–fully disagree to 5–fully agree. Seven items are reverse coded. The scale total score, which can range from 1 to 5, is the mean score of responses to all items. Higher scores on the CRAS-S reflect higher pro-animal attitudes. The psychometric properties of the CRAS-S are sound, and details can be found in Randler et al. [24]. Participants also indicated their eating preference and rated themselves as: 1 = omnivore, 2 = pescatarian, 3 = vegetarian, 4 = vegan.

To consider the influence of a country’s social and economic development, we used the Human Development Report [27] and extracted the gender development index (GDI) and the gender inequality index (GII). The GDI measures disparities on the Human Development Index (HDI) by gender. The Human Development Index (HDI) measures a country’s overall achievement on social and economic dimensions based on the health of people, their level of education attainment and their standard of living [27]. HDI values are estimated separately for women and men; the GDI value is the ratio of the HDI value for women and men. The closer the ratio is to 1, the smaller the gap between women and men. The gender inequality index (GII) is based on indicators (reproductive health, the proportion of women in the state parliament, completion of secondary education and labor market participation; [28]). A low GII value indicates low inequality between women and men, and vice-versa. Thus, both indices are negatively related with each other (in our sample of the 22 countries it is −0.800, *p* < 0.01).

### 2.3. Ethical Considerations

Ethical clearance to collect the data was first granted from the Ethik-Kommission at the University of Education Heidelberg (Az 7741.35-13). This ethical clearance was accepted in a translated form by other universities. In addition, clearance was also obtained from the Central Queensland University, Rockhampton, Australia (H15/11-263), Bishop University, Québec Canada (2015-30). The Bioethics Committee of the University of Bologna (Bologna, Italy) approved the study in Bologna. Ethical approval was obtained from the School of Psychology Human Ethics Committee (#22835, approval date 16 May 2016) before data were collected.

### 2.4. Statistical Analyses

#### 2.4.1. Measurement Invariance

Despite mapping distinct aspects, the CRAS-S was operationalised as a one-factor measure [24]. Before mean comparisons were performed, we examined the measurement invariance of the CRAS-S across the 24 samples and gender using the alignment method in Mplus version 7.4 [29]. We examined the dimensionality of the scale with exploratory factor analysis and confirmatory factor analysis before conducting multi-group analysis and examining the measurement invariance of the scale. All analyses are reported in the Supplementary Material. 

#### 2.4.2. General Linear Model

To assess country differences in CRAS-S score we applied a general linear model. The independent variables were gender, residence and diet, and age was used as a covariate. The total CRAS-S score was used as dependent variable. For the interpretation of eta-squared, we used Richardson’s approach [30].

#### 2.4.3. Meta-Analysis 

For the country analysis of gender effect, we calculated effect sizes using Comprehensive Meta-Analysis [31]. Compared to calculating mean differences across the entire sample, the meta-analytical approach is more sophisticated because every sample is treated independently, and the calculation of effect sizes takes into account the sample size. We used Hedge’s g as a measure of effect size. After converting mean sample differences into effect sizes, we used meta-regression in CMA to regress effect sizes against the two measures of GDI and GII.

#### 2.4.4. Further Analyses

We used SPSS 26 (IBM Corp., Armonk, NY) to calculate Cronbach’s alpha for the CRAS-S in every sample and in total and for the general linear model.

## 3. Results

Mean age across the sample was 23.91 years (SD = 8.1). Women were 65% of the participants, men were 34% of the sample and 1% did not answer. Our goal of gender balance was not achieved since many participants were drawn from disciplines that typically attract women candidates (e.g., psychology, education, and biology). A detailed breakdown of gender by country (and sample size) can be found in Table 1. The mean response to the CRAS-S was 3.51 (SD = 0.59) indicating a general tendency for pro-animal attitudes (scale ranged from 1–5). The highest mean score was reported in Colombia (3.87; SD = 0.47) and lowest score was in Iran (3.02; SD = 0.51). The adjusted means for CRAS-S after controlling for age, gender and diet can be found in Table 1. Scale reliability across all countries was high (0.82) suggesting the CRAS-S appears to be internally reliable across different languages in different regions of the world. Finland reported highest scale reliability (0.92) and the lowest was found in the Hindi sample from India (0.58) and Mozambique (0.60). In all samples, items correlated positively with the total scale, only one item loaded negatively on the scale in Mozambique (item 11; −0.09) and in the Hindi version in India (item 8; −0.008). As detailed in the Supplementary Material, measurement invariance was supported, and mean comparisons are thus meaningful.

**Figure 2:**
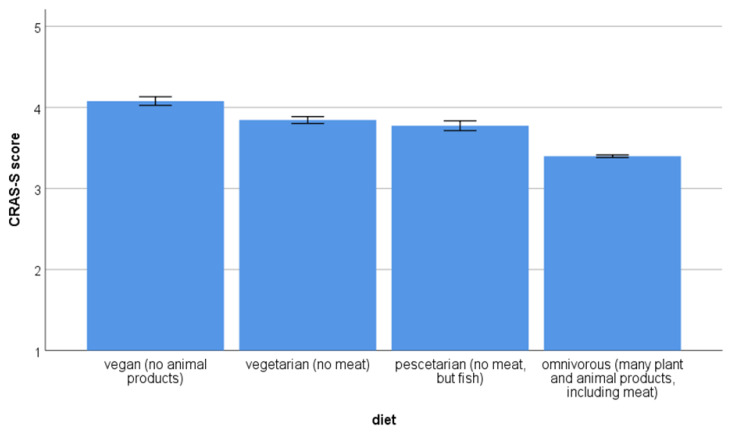
Animal welfare attitudes according to diet. Higher scores represent higher pro-animal welfare attitudes. Animal welfare attitudes (AWA) were measured with the Composite Respect for Animals Scale-Short Version (CRAS-S).

In terms of diet preference, 6385 participants reported to be omnivores, 296 were pescatarian, 637 were on a vegetarian diet and 434 were vegans (*n* = 162 without answer). Diet was related to AWA (Figure 2), that is, scores were higher for people on a vegan diet. In the case of omnivores, these participants showed the lowest values, below pescatarians and vegetarians. Post-hoc tests revealed statistically significant differences among all comparisons (*p* < 0.001) except for the difference between the pescatarian and vegetarian diets (0.056).

**Figure 3:**
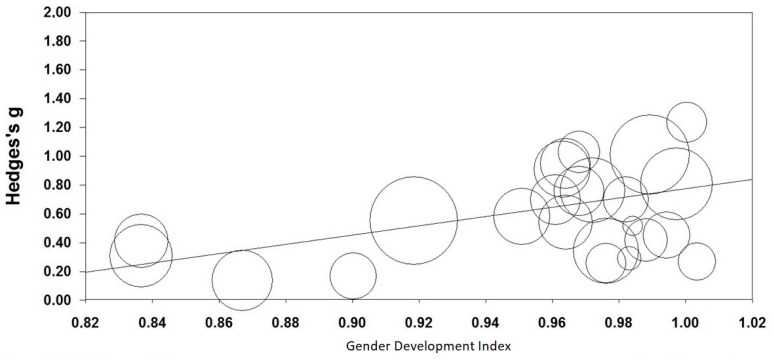
Relationship between gender development index and CRAS-S (AWA) scores. Higher scores of Hedge’s g represent a larger gender difference in the CRAS-S score and higher scores in Gender Development Index represent higher gender equality. Animal welfare attitudes (AWA) were measured with the Composite Respect for Animals Scale-Short Version (CRAS-S). Circles represent sample sizes.

In the meta-analysis using CMA [31], GDI was positively correlated with effect sizes (Slope = 3.25, z = 6.31, *p* < 0.001; *Q*_total_ = 122.03, df = 23, *n* = 24, *p* < 0.001; Figure 3). Effect sizes were greater in countries with smaller gap between women and men regarding human development.

**Figure 4:**
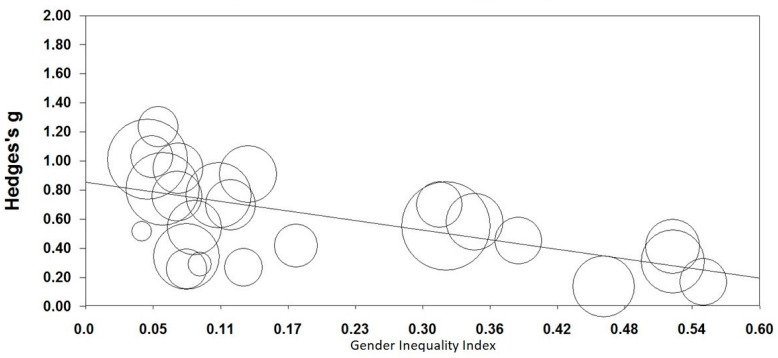
Relationship between gender inequality index and CRAS-S (AWA) scores. Higher scores of Hedge’s g represent a larger gender difference in the CRAS-S scores and higher scores in Gender Inequality Index represent lower gender equality. Animal welfare attitudes (AWA) were measured with the Composite Respect for Animals Scale-Short Version (CRAS-S). Circles represent sample sizes.

Similarly, GII was negatively related with effect sizes (Slope = −1.076, z = −7.34, *p* < 0.001; *Q*_total_ = 122.03, df = 23, *n* = 24, *p* < 0.001; Figure 4). This negative effect indicates effect sizes were greater in countries with low gender inequality.

## 4. Discussion

In this study, we confirmed and replicated gender differences in AWA, with women reporting a higher pro-animal attitude. Also, diet was related with AWA in an expected manner with vegan people reporting higher pro-animal attitudes. Concerning measurement, our study is advantageous in the sense that we administered the same psychometrically sound questionnaire, spanning a broad scope over many constructs, to all participants irrespective of the languages they used and the continents they live in. The statistical approach following this study is certainly superior to a regular meta-analysis, where usually results from different questionnaires were combined into a single score. In this respect, our meta-analysis approach was based on similar research tools, which enhances the validity of the research outcome. Although we received some support for such a measurement invariance by the alignment method, the meta-analytical approach is a more conservative one. In addition, some items were poorly correlated with the total scale score in three countries. However, we decided not to delete items from the scale to maintain the scale’s integrity, i.e., to keep all 20 items in the scale because in previous studies based on the scale [24] all items showed a good item-scale correlation. Nevertheless, it is important for future studies to refine the items and to further address and improve the invariance of the scale.

Concerning gender differences, our findings corroborate the results published earlier [2,4]. Different reasons and explanation have been forwarded to explain the gender differences in AWA from a psychological viewpoint: (1) males might have been evolved/socialized to be more utilitarian and less emotional, whilst females might have been socialized to care and nurture; (2) from an evolutionary standpoint, males were more involved in hunting than females, therefore, they are most likely to consider animals as potential food sources [5,32,33]. The first explanation is more related to female social roles, and thus, should be open for changes and modifications throughout shorter timespans, because social roles change more quickly compared to evolutionary changes. The evolutionary psychology approach suggests that observed patterns should be more or less stable in evolutionary times and be more oriented towards fitness, which means reproductive success [34]. Our data suggest a more social role explanation without strictly excluding evolutionary psychological aspects, because we found a relationship between gender equity/inequality and gender differences in AWA. Therefore, gender empowerment might also play a role [11]. In countries where females are more empowered, women express greater concern for animal welfare issues than men, whereas in other countries the responses of males and females were more or less similar. At the same time, this interpretation is somehow counterintuitive, e.g., assuming an evolutionary basis and a social-role transition would rather suggest that gender differences should disappear with higher empowerment of women [11]. However, as the study of Phillips et al. [11] and ours point into the same direction, we consider the findings valid, although further studies are needed to back-up these assumptions. One interpretation could be that greater empowerment provides an opportunity for women to develop and express their own attitudes with less social constraints [11], which leads to a larger gender difference in more gender empowered countries. Gender differences have been well established by Herzog [4], and most studies have found these effects regardless of country or culture. It would be an interesting aspect to further sample people from the urban–rural gradient, e.g., to compare city-dwellers with inhabitants from rural areas [35].

We found diet an important statistical predictor of AWA. This is expected because both may be correlated with each other [15,19,20]. The correlation gives some concurrent validity for the CRAS-S. Further, diet was used as a control for gender differences, because women, more than men, tend to become vegetarians. Thus, we disentangled the effect of gender and diet by using a multivariate model. Higher AWA and ethical concerns about the treatment of animals may be a reason for an individual to follow a vegetarian or a vegan diet, which can also be related to a specific religion [36]. However, being a vegetarian without caring for animal ethics may also be possible when vegetarianism develops in a person because of health concerns. The third reason, environmental concern and ecological aspects of meat consumption are poorly studied and research reports on the issue are either scarce or sometimes even absent [37,38]; however, a recent study has shown that environmental attitudes and AWA are correlated [39]. It would be beneficial in further studies on a longitudinal basis to look for cause and effect in the relationship between diet and AWA, to disentangle whether changes in attitude occur before changes in diet (because attitude change is easier to do than behavioral change).

There were no age effects in our study. This finding is in stark contrasts to studies that included both adolescents [15] and adult participants [13]. Our study, however, focused on university students and not on the general public, thus the age range of our respondents was rather narrow. Therefore, age effects were no expected in this study, but age was still incorporated as a covariate. In addition to age, other demographical and individual differences factors are related to AWA [2], e.g., field of study. As there is also important within-country cultural variation, these aspects should have been addressed in our study, e.g., concerning religion, language, and socio-economic status. As this study has one item labelled “I would like to be a veterinarian”, some of the students may already have chosen their profession, and it could be misunderstood. This should be considered in further refinements of the scale, e.g., such item could be replaced by “If I had a choice, I would prefer being a veterinarian”.

The study has strengths that include that the same psychometrically sound questionnaire was used throughout in a very large sample of university students from 22 countries. Limitations are the self-report nature of the study as well as the convenience sampling of university students, mainly from around university cities and from different subjects. The gender balance has not been fully achieved: women represented 66% of the total. However, the fact that women respond in greater numbers than men may already be an indication of their greater interest in the subject. Nevertheless, we cannot guarantee the representativeness of the gender distribution for all study locations due to the convenience sampling (which is sometimes female-biased), and the topic of the study (AWA), that may also rise more interest among women. Thus, future work should include a representative population sample to better match the gender differences on the micro-level with the HDI on the macro-level. However, most of the representative large cross-country samples allow only one or a few questions for such projects. Concerning culture, there are also differences within a country, so a given city or area cannot be representative for the whole country because of well-known differences between center and periphery [40]. Therefore, country differences should not be overinterpreted. In addition, European countries made up the majority of the data and there were only a few low-income countries from the Global South.

Anyway, despite a growing interest in consumer attitudes toward animal welfare in the developing markets like Latin America, most of the studies addressing consumer attitudes toward animal welfare have originated in Europe and North America. “Until now, Latin American consumers’ attitudes towards animal welfare have not been well studied” ([41], p. 697). Latin America covers a vast range of territories with varying geographical features and distinctive socio-economic, cultural, and political systems. These marked differences, in the view of some researchers, are reflected on the divergent opinions that citizens from this region have about animal welfare. Some studies argue that Latin American citizens are becoming more aware of and interested in animal welfare driven by a concern about the quality of food items derived from animals [42,43]. Others suggest that this phenomenon can be explained by high accessibility to goods and commodities of new generations, which seems to translate into new expectations regarding animal welfare [44]. This growing interest in animal welfare has encouraged the introduction of specific rules and practices in the production of red meat, a significant sector of the economy in many countries of the region [45].

In any case, this imbalance between European and low-income countries from the Global South should be addressed in future work also because such a bias towards Western, educated, industrialized, rich and democratic (WEIRD) societies has been criticized earlier [46]. 

## 5. Conclusions

The aim of this study was to explore gender differences in AWA examining a large sample of university students from 22 countries. The main take home message of this work is that women refer higher AWA than men. It is noteworthy that gender differences in AWA are higher in countries with lower of gender inequality, leading to suggest that gender empowerment could play a role in the modulation of human attitudes towards the welfare of animals. One interesting and important aspect for the future would be to focus less on differences between humans or individuals and what divides people (e.g., gender, eating style, lifestyle), but pay attention to what can connect people together. For further studies it might be worthwhile starting to analyze the similarities and levels of communication in the human-human and human-animal relationship.

## Figures and Tables

**Table 1:** Overview over the countries, including sample sizes, gender, age and reliability. Mean scores of the Composite Respect for the Composite Respect for Animals Scale Short version (CRAS-S) are also reported with high values representing high pro-animal attitude. The alpha refers to the unadjusted CRAS-S means. The GDI value is the ratio of the HDI value for women and men. The closer the ratio is to 1, the smaller the gap between women and men. A higher gender inequality index (GII) is related to a higher inequality.

Sample	Female (*n*)	Male (*n*)	No Answer	Total	Method	Mean Age	SD	Alpha	CRAS-S Mean Unadjusted	SD	d	Var(d)	GDI	GII
Germany	262	76	0	338	web based	24.49	4.58	0.832	3.65	0.52	0.94	0.02	0.967	0.072
Austria	322	69	0	391	web based	23.41	6.36	0.822	3.73	0.52	0.78	0.02	0.971	0.071
Switzerland	74	10	0	84	web based	23.17	5.31	0.857	3.61	0.54	0.53	0.12	0.987	0.039
Catalonia (Spain)	225	164	0	389	paper-pencil	22.61	2.14	0.840	3.70	0.56	0.35	0.01	0.979	0.080
Australia	299	146	0	445	web based	40.22	15.10	0.895	3.44	0.71	0.76	0.01	0.975	0.109
Canada	27	22	1	50	both	23.45	8.78	0.884	3.53	0.61	0.28	0.08	0.986	0.092
New Zealand	224	116	5	345	web based	19.03	2.96	0.863	3.63	0.59	0.90	0.01	0.966	0.136
Turkey	539	259	10	808	paper-pencil	19.85	2.06	0.720	3.40	0.47	0.54	0.01	0.922	0.317
Spain	50	119	0	169	paper-pencil	20.78	4.05	0.850	3.58	0.59	−0.26	0.03	0.979	0.080
Sweden	556	211	5	772	web based	30.99	10.00	0.800	3.56	0.53	1.02	0.01	0.992	0.044
Slovenia	194	49	0	243	paper-pencil	20.31	1.80	0.821	3.47	0.49	1.21	0.03	1.003	0.054
Slovakia	295	47	0	342	web based	20.46	4.21	0.796	3.28	0.53	0.41	0.02	0.991	0.180
Romania	116	82	3	201	paper-pencil	21.17	1.83	0.759	3.40	0.45	0.70	0.02	0.985	0.311
Colombia	260	60	1	321	paper-pencil	21.61	3.48	0.780	3.87	0.47	0.44	0.02	0.997	0.383
Poland	200	37	0	237	web based	26.46	6.94	0.895	3.50	0.67	0.27	0.03	1.006	0.132
Mozambique	75	125	0	200	paper-pencil	26.59	7.19	0.598	3.31	0.42	−0.19	0.02	0.904	0.552
Italy	279	85	2	366	paper-pencil	20.76	2.41	0.867	3.58	0.58	0.53	0.02	0.967	0.087
Mexico	177	123	0	300	paper-pencil	19.82	1.84	0.820	3.73	0.50	0.58	0.01	0.954	0.343
India (Hindi)	238	137	0	375	paper-pencil	22.50	2.57	0.575	3.68	0.43	0.29	0.01	0.841	0.524
Finland	253	247	0	500	web based	25.72	7.25	0.919	3.32	0.78	0.81	0.01	1.000	0.058
Belgium/Flandern	88	88	18	194	paper-pencil	21.07	8.29	0.892	3.35	0.66	−1.03	0.03	0.971	0.048
India (English)	143	117	0	260	paper-pencil	22.09	1.20	0.596	3.38	0.47	0.40	0.02	0.841	0.524
Iran	170	156	0	326	paper-pencil	21.66	3.47	0.770	3.02	0.51	0.12	0.01	0.871	0.461
Greece	89	166	3	258	paper-pencil	20.96	4.99	0.745	3.58	0.51	−0.69	0.02	0.964	0.120
**Total sample**	**5155**	**2711**	**48**	**7914**		**23.91**	**8.10**	**0.817**	**3.51**	**0.59**				

Values in bold are refed to the total sample.

**Table 2:** Results of a general linear model with CRAS-S score as dependent variable, gender, residence, and diet as fixed factors, and age as covariate. MS = mean of squares, partial eta^2^ = explained variance.

Source of Variance	df	MS	F	*p*	Partial eta^2^
Corrected model	28	21.648	82.466	<0.001	0.232
Constant	1	5879.542	22,397.105	<0.001	0.746
Gender	1	86.838	330.796	<0.001	0.041
Residence	23	9.720	37.027	<0.001	0.100
Diet	3	83.260	317.166	<0.001	0.111
Age	1	0.087	0.331	0.565	0.000
Error	7644	0.263			
Total	7673				

## Data Availability

The associated data and the questionnaires are available on the Open Science Framework under https://osf.io/q2dh7/ (accessed on 28 May 2021).

## Supplementary Materials

The following are available online at https://www.mdpi.com/article/10.3390/ani11071893/s1, Table S1: Approximate measurement invariance (noninvariance) with the alignment method of the CRAS-S scale over 24 samples. Table S2: Alignment fit statistics for the CRAS-S scale across 24 samples. Table S3: Factor mean comparisons of the CRAS-S scale across 24 groups based on measurement invariance with alignment method. Table S4: Approximate measurement invariance (noninvariance) with the alignment method of the CRAS-S scale over gender. Table S5: Alignment fit statistics for the CRAS-S scale across gender. Supplementary Section A: Scree plot with parallel analysis using 200 random samples form an exploratory factor analysis using the default Mplus setting (i.e., maximum likelihood estimation and geomin rotation) for the whole sample (*n* = 7890). Supplementary Section B: Results of the exploratory factor analysis of the CRAS-S Scale setting 1 to 2 factors.

## Appendix A. Items of the CRAS-S (24)

**Table A1:** Items of the Composite Respect for Animals Scale (short version).

As long as adequate food, warmth and light are provided, there is nothing really cruel about battery hen farming.
It is wrong to kill crocodiles to make shoes and handbags from their skins.
I would like to be a veterinarian.
It is acceptable to test cosmetics/shampoos on animals, so that they will not harm humans.
There is nothing morally wrong with hunting wild animals for food.
In my opinion, animals are definitely inferior to humans.
All insects should be protected.
I think it is perfectly acceptable for animals to be raised for human consumption.
I find my pet a source of emotional comfort (or would if I had one).
It is wrong to keep animals in zoos.
I do not think that there is anything wrong with using animals in medical research.
Angling is cruel and inhumane to the animals.
It is wrong to kill animals to make fur coats.
It is wrong to keep chickens in battery cages.
I do not believe that humans are superior to animals.
I would like to spend some of my time telling people about the problems that face an endangered animal.
Hunting helps people appreciate natural processes.
All animals should be conserved.
It is wrong to use animals in circuses.
I think of my pet as a member of my family (or would if I had one).
