# Supplementary material for: Animal Welfare Attitudes: Effects of Gender and Diet in University Samples from 22 Countries
Source: Animals (Basel). 2021 Jun 25;11(7):1893. doi: 10.3390/ani11071893 (PMC8300362; doi:10.3390/ani11071893)
Supplement: Supplementary file 1 [file animals-11-01893-s001.zip › animals-1270122-supplementary.pdf]

Measurement Invariance of the Composite Respect for Animals Scale Short version (CRAS-S Scale)

As detailed in the main document, the Composite Respect for Animals Scale Short version (CRAS-S Scale) is a tool to assess distinct dimensions of Animal Welfare Attitudes, from utilitarian aspects such as using animals for food, clothing, recreation, and research to concerns many individuals have about pets, conservancy of species, and attitudes of superiority over animals. Despite mapping distinct aspects, the CRAS-S was operationalized as a one-factor measure. We examined the dimensionality of the scale with exploratory factor analysis and confirmatory factor analysis before conducting multi-group analysis and examining the measurement invariance of the scale. All analyses reported in this Supplementary Material were conducted in Mplus version 7.4.

We first conducted exploratory factor analysis using the default Mplus setting (i.e., maximum likelihood estimation and geomin rotation) for the pooled-within covariance matrix. Parallel analysis with 200 random samples indicates five is the optimum number of factors, but the scree plot indicates that a two-factor structure is a much more parsimonious solution (see Appendix A). Exploratory factor analysis results indicated that the two-factors solution [$\chi2$ (N = 7914, df = 151) = 4180.537, p < .001; RMSEA = .058 [90% CI = .057, .060]; CFI = .907; SRMR = .035] was better fitting to the data than a one-factor [$\chi2$ (N = 7914, df = 170) = 8156.196, p < .001; RMSEA = .077 [90% CI = .076, .079]; CFI = .816; SRMR = .064]. However, inspection of the item loadings clearly indicates the two-factors solution is not very clear (see Appendix B).

To provide further evidence of the one-factor structure of the CRAS-S, we conducted a confirmatory factor analysis for the whole sample using maximum likelihood estimation with robust standard errors. Since some of the CRAS-S items are negatively worded, we modelled common-method bias with a method factor fixing loadings of positively worded items to +1 and negatively worded items to -1 for this factor, while allowing items loadings to be freely estimated for the content CRAS-S factor and fixing the content and method factor correlation to zero. The fit of this model to the data was reasonable: S-B$\chi2$ (N = 7914, df = 169) = 11424.750, p < .001; *RMSEA = .092 [90% CI = .090, .093]; *CFI = .601; *SRMR = .078. Allowing ten error terms with highest modification indices (i.e., greater than 200) improved the fit: S-B$\chi2$ (N = 7914, df = 159) = 4098.838, p < .001; *RMSEA = .056 [90% CI = .054, .057]; *CFI = .860; *SRMR = .053.

The results reported above provide support for treating the CRAS-S as a single factor tool. We proceed by examining the measurement invariance of the CRAS-S scale with the alignment optimization method. The goal of the alignment method is to correctly estimate the ordering of groups with respect to factor means (Asparouhov & Muthén, 2014; Muthén & Asparouhov, 2018), and it is ideal for testing measurement invariance for large number of groups (Byrne, & van de Vijver, 2017).

We selected the FREE alignment estimates as the first analytical step, using the FIXED alignment estimates if required (see Asparouhov & Muthén, 2014). The alignment method does not provide standard model fit indicators. Following past research (Asparouhov & Muthén, 2014; Byrne, & van de Vijver, 2017), we examined the fit function contribution and the R^2 statistics. The fit function contribution captures the amount of noninvariance from each parameter for each item variance, with lower values representing higher degrees of noninvariance. The R^2 statistics measures the degree of noninvariance that can be absorbed by group-varying factor means, with values closer to 1 representing higher degrees of noninvariance.

We conducted alignment analyses separately for cultural groups and gender, and we detail the procedures and results below. Overall, the results indicate that not all items from the CRAS-S scale are invariant across all cultural groups and gender. At the same time, Muthén and Asparouhov (2018) showed that it is not necessary for all items to have invariance measurement parameters across all groups to obtain good alignment performance. We also employed Monte Carlo simulations to check the quality of the solutions obtained from the alignment method.

The simulation goal is to estimate the ordering of the groups for the factor means obtained, with correlation values indicating the success of the alignment method for obtaining measurement invariance. We used 500 replications and varied the group sample sizes to n = 200 and n = 500. The simulated correlations between generated and estimated factor means for these estimated sample sizes was 1.00 for both factor mean and factor variance when comparing gender. The simulated correlations between generated and estimated factor means were also very high when comparing the 24 samples for both n = 200 (r = .998 for factor mean; r = 0.985

for factor variance) and n = 500 (r = .999 for factor mean; r = .986 for factor variance). These simulation results confirm the trustworthiness of aligned scales and results detailed below and provide support for the measurement invariance of the CRAS-S scale (see Muthén & Asparouhov, 2018). Based on the alignment results, the measurement invariance of the CRAS-S was deemed acceptable and mean comparisons are thus meaningful.

Alignment results across cultural groups

The FREE alignment model examining measurement invariance of the CRAS-S scale across cultural groups produced a warning of untrustworthy standard errors. We then used the FIXED approach by using New Zealand as the reference group with a latent factor mean of 0, because this group had the closest latent mean of 0 in the FREE model.

Table S1 presents noninvariance information for both factor loadings and item intercepts by country based on the final FIXED model. As common, there are more noninvariant item intercepts than noninvariant factor loadings. Further inspection indicates that the intercepts of item 20 (i.e., *I think of my pet as a member of my family (or would if I had one)*) is invariant in almost all countries. This is confirmed in Table S2 that provides the fitting functions of both the factor loading and intercept for each item. Item 20 has the lowest overall contribution to the fitting function (-349.31). Table S5 presents the latent mean estimates and their comparisons across the 24 samples. The correlation between the latent score and the raw score (averaging over items after reverse coding relevant items) of the CRAS-S scale was high (r = .887, p < .001, N = 7914). Together with the Monte Carlo simulation results reported above, these results give us confidence in the trustworthiness of the latent mean estimates and alignment results.

Please note that an error message was present in the alignment analysis across cultural groups. It stated that the standard errors of the model parameter estimates may not be trustworthy for some parameters due to a non-positive definite first-order derivative product matrix. Most likely it was related to item 1.

Table S1. Approximate measurement invariance (noninvariance) with the alignment method of the CRAS-S scale over 24 samples

Item	Country
Factor Loadings	
CRAS1	(1) (2) 3 (6) 7 8 11 12 13 14 15 16 17 18 19 20 (21) 22 (23) (24) (25) (26) 27 31
CRAS2	1 2 3 (6) 7 8 11 12 13 14 (15) 16 17 18 19 20 (21) 22 23 24 25 (26) 27 31
CRAS3	1 (2) 3 6 7 8 11 12 13 (14) 15 16 17 18 19 20 21 22 23 (24) 25 26 27 31
CRAS4	1 2 3 6 7 8 11 12 13 (14) 15 16 17 18 19 20 21 22 (23) 24 25 (26) 27 31
CRAS5	(1) 2 3 6 7 8 11 (12) 13 14 15 (16) (17) 18 (19) 20 21 22 23 24 25 (26) 27 31
CRAS6	1 2 3 6 7 8 11 (12) 13 14 15 (16) 17 18 19 20 (21) 22 23 24 25 (26) 27 (31)
CRAS7	1 2 3 6 7 8 11 12 13 14 15 16 17 18 19 20 21 22 23 24 25 26 27 31
CRAS8	1 (2) 3 6 7 8 (11) (12) 13 14 15 16 17 18 19 20 21 22 (23) (24) 25 (26) 27 31
CRAS9	1 2 3 6 (7) 8 11 12 13 (14) (15) 16 17 18 (19) 20 21 22 23 (24) 25 26 27 31
CRAS10	1 2 3 6 7 8 11 12 13 14 15 16 17 18 19 20 (21) 22 23 24 25 26 27 31
CRAS11	1 2 3 6 7 8 11 12 13 14 15 16 17 18 19 (20) 21 22 (23) 24 25 (26) 27 31
CRAS12	(1) (2) 3 6 7 8 11 12 13 14 15 16 17 18 (19) 20 21 22 23 24 25 (26) 27 31
CRAS13	(1) (2) (3) (6) 7 8 11 12 13 14 (15) 16 17 18 19 20 (21) 22 23 24 (25) (26) 27 31
CRAS14	(1) (2) 3 6 7 8 (11) 12 13 (14) 15 16 17 18 19 20 21 22 23 (24) (25) (26) 27 31
CRAS15	1 2 3 6 7 8 11 (12) 13 14 15 16 17 18 19 20 21 22 23 24 25 (26) 27 31
CRAS16	1 2 3 6 7 8 11 12 13 14 15 16 17 18 19 20 21 22 23 24 25 (26) 27 31
CRAS17	1 2 3 6 7 8 11 (12) 13 14 15 16 17 18 19 20 21 22 23 24 25 (26) 27 31
CRAS18	1 2 3 6 7 8 11 12 13 (14) 15 16 17 18 (19) 20 21 22 23 24 25 26 27 31
CRAS19	(1) 2 3 (6) 7 8 11 12 13 14 15 16 17 18 (19) 20 (21) (22) 23 24 25 (26) 27 31
CRAS20	1 (2) 3 6 (7) 8 (11) 12 13 14 15 16 17 (18) 19 20 21 22 23 (24) 25 (26) 27 31
Item Intercepts	
CRAS1	(1) (2) (3) (6) (7) 8 (11) (12) (13) 14 15 (16) 17 18 (19) 20 21 (22) (23) (24) (25) (26) 27 31
CRAS2	1 2 3 6 (7) 8 11 12 13 14 (15) 16 17 18 19 20 21 (22) 23 24 25 26 27 31
CRAS3	(1) (2) (3) (6) 7 8 11 12 (13) 14 15 (16) (17) 18 (19) (20) (21) 22 23 (24) 25 26 27 31
CRAS4	(1) (2) (3) 6 (7) 8 (11) 12 13 (14) (15) (16) 17 18 19 20 21 22 (23) (24) 25 26 27 31
CRAS5	(1) (2) 3 (6) (7) (8) (11) 12 13 (14) 15 (16) (17) 18 19 (20) (21) 22 (23) (24) 25 26 (27) 31
CRAS6	1 2 3 (6) 7 8 11 (12) 13 (14) (15) 16 17 (18) 19 20 21 22 (23) (24) 25 26 27 31
CRAS7	1 2 3 6 7 8 11 12 (13) (14) (15) (16) 17 (18) (19) (20) 21 22 (23) (24) 25 26 27 31
CRAS8	1 2 3 (6) (7) 8 11 (12) (13) (14) (15) (16) 17 18 19 20 21 22 (23) 24 25 26 27 (31)
CRAS9	1 (2) 3 6 (7) (8) (11) (12) 13 (14) 15 16 (17) 18 (19) 20 (21) (22) 23 (24) 25 26 27 (31)
CRAS10	1 2 3 (6) (7) 8 11 12 (13) (14) 15 16 17 18 19 20 (21) 22 23 24 25 26 27 31
CRAS11	(1) (2) (3) 6 (7) 8 (11) (12) 13 14 (15) (16) (17) 18 (19) (20) (21) (22) (23) 24 25 26 27 31
CRAS12	1 2 3 6 (7) 8 11 (12) (13) 14 15 (16) 17 18 (19) (20) (21) (22) (23) (24) 25 (26) 27 (31)
CRAS13	1 2 3 6 (7) 8 (11) 12 13 14 15 16 (17) (18) 19 20 21 (22) 23 24 25 26 27 31
CRAS14	(1) (2) (3) 6 7 8 (11) 12 13 14 (15) 16 (17) (18) 19 20 21 (22) (23) 24 25 (26) 27 (31)
CRAS15	1 2 3 (6) (7) 8 11 (12) 13 14 (15) 16 17 18 19 20 21 22 23 (24) (25) 26 27 31
CRAS16	1 2 3 6 (7) (8) 11 12 13 14 (15) 16 (17) 18 19 (20) 21 (22) (23) 24 25 (26) (27) (31)
CRAS17	1 2 3 (6) 7 8 (11) (12) (13) (14) 15 16 17 (18) (19) 20 21 22 23 (24) 25 26 27 (31)
CRAS18	1 2 3 6 7 8 11 12 13 14 (15) (16) 17 (18) (19) (20) (21) (22) (23) 24 25 26 (27) (31)
CRAS19	(1) (2) 3 (6) 7 8 11 12 (13) (14) 15 (16) 17 18 (19) 20 (21) (22) 23 24 25 26 27 (31)
CRAS20	1 2 3 6 7 8 11 (12) 13 14 15 16 17 18 19 20 21 22 23 24 25 26 27 31

Note. Noninvariant parameters are parenthesized. Sample numbers are: 1=Germany, 2=Austria, 3=Switzerland, 6=Spain (Catalan), 7=Australia, 8=Canada, 11=New Zealand, 12=Turkey, 13=Spain (Spanish), 14=Sweden, 15=Slovenia, 16=Slovakia, 17=Romania, 18=Colombia, 19=Poland, 20=Mozambique, 21=Italy, 22=Mexico, 23=India (Hindi), 24=Finland, 25=Belgium (Flanders), 26=India (English), 27=Iran, 31=Greece

Table S2. Alignment fit statistics for the CRAS-S scale across 24 samples

		Factor loadings		Intercepts		Loadings + Intercepts
Item Code and #	Item wording	Fit function contribution	R^2	Fit function contribution	R^2	Total contribution
CRAS1	As long as adequate food, warmth and light are provided, there is nothing really cruel about battery hen farming.	-193.64	.217	-302.02	.000	-495.66
CRAS2	It is wrong to kill crocodiles to make shoes and handbags from their skins.	-177.33	.124	-210.07	.186	-387.40
CRAS3	I would like being a veterinarian.	-178.66	.092	-277.26	.000	-455.92
CRAS4	It is acceptable to test cosmetics/shampoos on animals, so that they will not harm humans.	-157.53	.075	-217.03	.000	-374.56
CRAS5	There is nothing morally wrong with hunting wild animals for food.	-182.93	.095	-272.20	.000	-455.13
CRAS6	In my opinion, animals are definitely inferior to humans.	-174.07	.011	-209.81	.000	-383.88
CRAS7	All insects should be protected.	-131.20	.391	-258.09	.283	-389.29
CRAS8	I think it is perfectly acceptable for animals to be raised for hu-man consumption.	-188.48	.157	-193.44	.000	-381.92
CRAS9	I find my pet a source of emotional comfort (or would if I had one).	-198.79	.000	-285.69	.189	-484.48
CRAS10	It is wrong to keep animals in zoos.	-144.17	.132	-217.86	.402	-362.03
CRAS11	I do not think that there is anything wrong with using animals in medical research.	-181.52	.138	-220.55	.000	-402.08
CRAS12	Angling/Fishing is cruel and inhumane to the animals.	-161.30	.158	-248.46	.167	-409.76
CRAS13	It is wrong to kill animals to make fur coats.	-180.83	.016	-175.29	.103	-356.12
CRAS14	It is wrong to keep chickens in battery cages.	-167.79	.241	-224.46	.000	-392.25
CRAS15	I do not believe that humans are superior to animals.	-158.37	.198	-212.86	.000	-371.24
CRAS16	I would like to spend some of my time telling people about the problems that face an endangered animal.	-139.49	.330	-262.88	.003	-402.37
CRAS17	Hunting helps people appreciate natural processes.	-181.11	.061	-237.53	.110	-418.64
CRAS18	All animals should be conserved/protected.	-153.25	.295	-262.44	.123	-415.70
CRAS19	It is wrong to use animals in circuses.	-154.97	.064	-196.98	.525	-351.94

CRAS20	I think of my pet as a member of my family (or would if I had one).	-167.65	.000	-181.66	.423	-349.31

Table S3. Factor mean comparisons of the CRAS-S scale across 24 groups based on measurement invariance with alignment method

Ranking	Latent Class	Group Value	Factor Mean	Groups with significantly smaller factor mean
1	India (English)	26	1.322	18 23 22 2 6 1 14 11 13 31 19 8 3 12 21 7 15 25 16 24 17 20 27
2	Colombia	18	0.569	23 22 2 6 1 14 11 13 31 19 8 3 12 21 7 15 25 16 24 17 20 27
3	India (Hindi)	23	0.275	31 19 12 21 7 15 25 16 24 17 20 27
4	Mexico	22	0.264	1 14 11 13 31 19 3 12 21 7 15 25 16 24 17 20 27
5	Austria	2	0.212	14 11 13 31 19 12 21 7 15 25 16 24 17 20 27
6	Spain (Catalan)	6	0.125	21 7 15 25 16 24 17 20 27
7	Germany	1	0.073	21 7 15 25 16 24 17 20 27
8	Sweden	14	0.027	7 15 25 16 24 17 20 27
9	New Zealand	11	0	7 15 25 16 24 17 20 27
10	Spain (Spanish)	13	-0.022	15 25 16 24 17 20 27
11	Greece	31	-0.037	7 15 25 16 24 17 20 27
12	Poland	19	-0.049	15 25 16 24 17 20 27
13	Canada	8	-0.051	25 16 24 17 20 27
14	Switzerland	3	-0.052	25 16 24 17 20 27
15	Turkey	12	-0.097	15 25 16 24 17 20 27
16	Italy	21	-0.127	25 16 24 17 20 27
17	Australia	7	-0.272	24 17 20 27
18	Slovenia	15	-0.324	17 20 27
19	Belgium (Flanders)	25	-0.442	20 27
20	Slovakia	16	-0.491	20 27
21	Finland	24	-0.551	20 27
22	Romania	17	-0.629	27
23	Mozambique	20	-0.925	
24	Iran	27	-1.189	

Note. Results from the FIXED approach with New Zealand as the reference group with a latent factor mean fixed to 0, because this group had the closest latent mean of 0 in the FREE model.

Alignment results across gender

The FREE alignment model examining measurement invariance of the CRAS-S scale across gender also produced a warning of untrustworthy standard errors. We then used the FIXED approach by using female participants as the reference group with a latent factor mean of 0, because this group had the closest latent mean of 0 in the FREE model. Table S4 presents noninvariance information for both factor loadings and item intercepts by gender. As common, there are more noninvariant item intercepts than noninvariant factor loadings, but 13 items showed to be noninvariant for factor loadings and item intercepts. Inspection of Table S5 indicates that item 6 (i.e., *In my opinion, animals are definitely inferior to humans*) had the lowest overall contribution to the fitting function (-0.66). Replicating results in the main article, male participants scored significantly lower on the CRAS-S scale (-0.574) compared to female participants (reference group with latent mean set to 0).

Table S4. Approximate measurement invariance (noninvariance) with the alignment method of the CRAS-S scale over gender

Item	Gender	Item	Gender
Factor Loadings		Item Intercepts	
CRAS1	1 2	CRAS1	1 2
CRAS2	1 2	CRAS2	1 2
CRAS3	1 2	CRAS3	1 2
CRAS4	1 2	CRAS4	1 2
CRAS5	1 2	CRAS5	1 2
CRAS6	1 2	CRAS6	1 2
CRAS7	1 2	CRAS7	(1) (2)
CRAS8	1 2	CRAS8	1 2
CRAS9	1 2	CRAS9	1 2
CRAS10	1 2	CRAS10	(1) (2)
CRAS11	1 2	CRAS11	(1) (2)
CRAS12	1 2	CRAS12	1 2
CRAS13	(1) (2)	CRAS13	1 2
CRAS14	1 2	CRAS14	1 2
CRAS15	1 2	CRAS15	(1) (2)
CRAS16	1 2	CRAS16	(1) (2)
CRAS17	1 2	CRAS17	1 2
CRAS18	1 2	CRAS18	(1) (2)
CRAS19	1 2	CRAS19	1 2
CRAS20	1 2	CRAS20	1 2

Note. Noninvariant parameters are parenthesized. Sample numbers are: 1=Female, 2=Male

Table S5. Alignment fit statistics for the CRAS-S scale across gender

		Factor loadings		Intercepts		Loadings + Intercepts
Item Code and #	Item wording	Fit function contribution	R^2	Fit function contribution	R^2	Total contribution
CRAS1	As long as adequate food, warmth and light are provided, there is nothing really cruel about battery hen farming.	-0.32	1.000	-0.44	.947	-0.75
CRAS2	It is wrong to kill crocodiles to make shoes and handbags from their skins.	-0.37	.740	-0.33	.990	-0.69
CRAS3	I would like being a veterinarian.	-0.54	.000	-0.63	.000	-1.17
CRAS4	It is acceptable to test cosmetics/shampoos on animals, so that they will not harm humans.	-0.37	.740	-0.42	.944	-0.79
CRAS5	There is nothing morally wrong with hunting wild animals for food.	-0.41	.631	-0.38	.978	-0.79
CRAS6	In my opinion, animals are definitely inferior to humans.	-0.34	.833	-0.32	1.000	-0.66
CRAS7	All insects should be protected.	-0.36	.000	-0.65	--	-1.01
CRAS8	I think it is perfectly acceptable for animals to be raised for human consumption.	-0.36	.000	-0.35	.992	-0.71
CRAS9	I find my pet a source of emotional comfort (or would if I had one).	-0.55	.000	-0.56	.919	-1.11
CRAS10	It is wrong to keep animals in zoos.	-0.32	.964	-0.49	--	-0.81
CRAS11	I do not think that there is anything wrong with using animals in medical research.	-0.33	.000	-0.52	--	-0.85
CRAS12	Angling/Fishing is cruel and inhumane to the animals.	-0.33	.000	-0.46	--	-0.79
CRAS13	It is wrong to kill animals to make fur coats.	-0.48	--	-0.32	.691	-0.80
CRAS14	It is wrong to keep chickens in battery cages.	-0.33	.878	-0.45	.991	-0.78
CRAS15	I do not believe that humans are superior to animals.	-0.32	1.000	-0.46	.935	-0.78
CRAS16	I would like to spend some of my time telling people about the problems that face an endangered animal.	-0.43	.000	-0.58	--	-1.02
CRAS17	Hunting helps people appreciate natural processes.	-0.43	.585	-0.40	--	-0.83
CRAS18	All animals should be conserved/protected.	-0.33	.304	-0.55	.944	-0.88
CRAS19	It is wrong to use animals in circuses.	-0.37	.746	-0.33	.998	-0.70

CRAS20	I think of my pet as a member of my family (or would if I had one).	-0.38		.000	-0.45		.954	-0.84

Note. Missing R^2 values indicates approximate Invariance was not found for this parameter.

References for supplementary material

Asparouhov, T., & Muthén, B. (2014). Multiple-group factor analysis alignment. *Structural Equation Modeling: A Multidisciplinary Journal*, *21*(4), 495–508. https://doi.org/10.1080/10705511.2014.919210

Byrne, B. M., & van de Vijver, F. J. R. (2017). The maximum likelihood alignment approach to testing for approximate measurement invariance: A paradigmatic cross-cultural application. *Psicothema, 29*(4), 539–551.

Muthén, B. & Asparouhov, T. (2018). Recent methods for the study of measurement invariance with many groups: Alignment and random effects. *Sociological Methods & Research, 2018*, *47*, 637–664. https://doi.org/10.1177/0049124117701488

Appendix for supplementary material

Appendix A. Scree plot with parallel analysis using 200 random samples form an exploratory factor analysis using the default Mplus setting (i.e., maximum likelihood estimation and geomin rotation) for the whole sample (*N* = 7890).

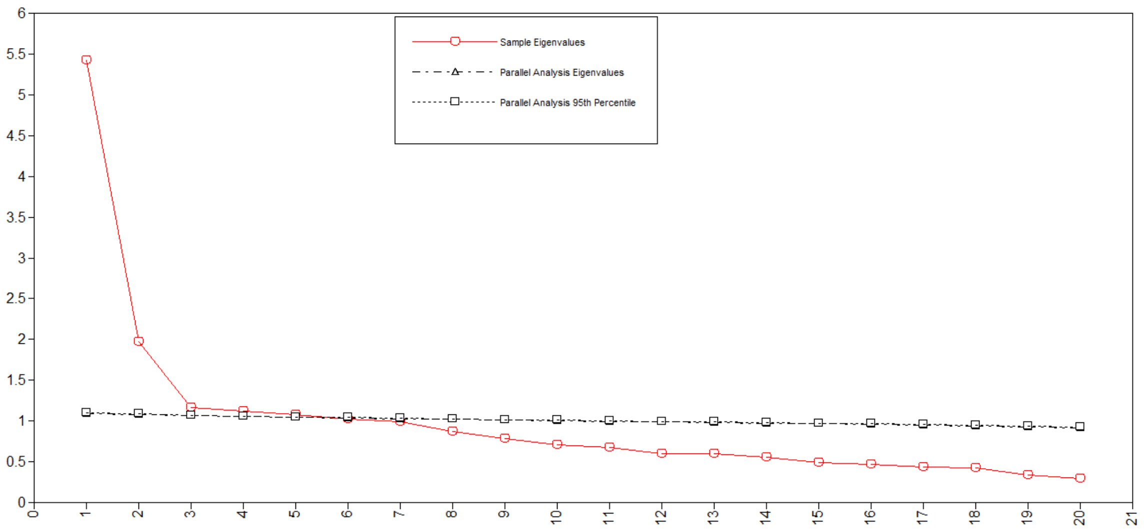

Appendix B. Results of the exploratory factor analysis of the CRAS-S Scale setting 1 to 2 factors.

EXPLORATORY FACTOR ANALYSIS WITH 1 FACTOR:

GEOMIN ROTATED LOADINGS (* significant at 5% level)

	1
CRAS1	0.012
CRAS2	0.026*
CRAS3	0.043*
CRAS4	0.015
CRAS5	0.056*
CRAS6	0.065*
CRAS7	0.014
CRAS8	0.067*
CRAS9	0.063*
CRAS10	0.095*
CRAS11	0.739*
CRAS12	0.626*
CRAS13	0.782*
CRAS14	0.610*
CRAS15	0.579*

CRAS16	0.737*
CRAS17	0.724*
CRAS18	0.698*
CRAS19	0.706*
CRAS20	0.765*

EXPLORATORY FACTOR ANALYSIS WITH 2 FACTOR(S):
GEOMIN ROTATED LOADINGS (* significant at 5% level)

	1	2
CRAS1	0.008	0.022
CRAS2	-0.022	0.331*
CRAS3	0.015	0.186*
CRAS4	0.015	0.003
CRAS5	-0.001	0.402*
CRAS6	-0.007	0.511*
CRAS7	-0.004	0.130*
CRAS8	0.023	0.320*
CRAS9	0.001	0.451*
CRAS10	0.040*	0.401*
CRAS11	0.712*	0.258*
CRAS12	0.614*	0.082*
CRAS13	0.766*	0.122*
CRAS14	0.594*	0.102*
CRAS15	0.571*	0.052*
CRAS16	0.751*	-0.093*
CRAS17	0.743*	-0.114*
CRAS18	0.696*	0.004*
CRAS19	0.721*	-0.099*
CRAS20	0.785*	-0.111*
